# Manifestations of Ollier's disease in a 21-year-old man: a case report

**DOI:** 10.1186/1752-1947-3-7759

**Published:** 2009-05-28

**Authors:** Babak Fallahi, Morteza Bostani, Kianoush Ansari Gilani, Davood Beiki, Ali Gholamrezanezhad

**Affiliations:** 1 Research Institute for Nuclear Medicine, Tehran University of Medical Sciences, Shariati Hospital, Northern Kargar Street, Tehran, Iran; 2 Young Researchers Club, Azad Medical Unit, Gholhak, Zargandeh, Tehran, Iran

## Abstract

**Introduction:**

Ollier's disease is a rare nonhereditary disorder characterized by multiple enchondromas with a predilection for unilateral distribution. Malignant changes in Ollier's disease may occur in adult patients. Radionuclide bone scanning is one method used to assess lesions depicted on radiographs or magnetic resonance images that are presumed to be enchondromas. Also, a bone scan may give a clue to the multifocal nature of the disease and it has been recommended that scintigraphy is useful in the monitoring of lesions and the development of any malignant transformation.

**Case presentation:**

A 21-year-old man with a history of pathologic fractures of the right tibia and multiple limb surgeries related to Ollier's disease was referred to our nuclear medicine department. Radiographic assessment showed multiple radiolucent expansile lesions, suggestive of multiple enchondromas. A whole-body bone (^99m^Tc-MDP) scan showed multiple foci of increased activity involving the proximal and distal right femur and tibia, proximal right humerus, distal right ulna, right metacarpals, metatarsals and phalyngeal tubular bones, consistent with unilateral distribution of the lesions. The long bones of the left hemi-skeleton were unremarkable, representing unilateral involvement of the skeleton. In this case, the intensity of uptake in the lesions of the lower extremity was high, raising the possibility of malignant degeneration of the enchondromas. Hence, the patient underwent surgical excision of the suspected lesions. Pathology analysis revealed their benign nature.

**Conclusion:**

Although the malignant transformation of enchondromas is a well known phenomenon, it should be kept in mind that other etiologies can be considered as the cause of intensely increased uptake. Retrospective assessment of our patient revealed that the etiology of increased uptake in the lower limb lesions was due to previous insufficiency fractures and the possibility of malignant transformation was ruled out based on the pathology findings.

## Introduction

Ollier's disease, a rare nonhereditary disorder characterized by multiple enchondromas with a predilection for unilateral distribution [1], was initially described by Ollier in 1899 [2]. The characteristic features of the disease are created by persisting cartilage masses in the metaphyses and diaphyses, which are formed by subperiosteal deposition of cartilage [2]. In fact, echondromas tend to occupy the diaphyseal region in the short tubular bones and the metaphyseal region in the long bones [1]. The pattern of limb involvement is usually asymmetrical, with one side being exclusively or predominantly involved [2].

The disease is usually detected during early childhood [3]. Notable clinical problems are progressive shortening of the involved extremity, angular deformity, pathological fractures and malignant transformation in 20% to 50% of cases [2,3]. There may be some gait problems caused by limb-length discrepancy, as lesions frequently involve the femur or tibia [2]. Malignant changes in Ollier's disease may occur in adult patients [2]. As dysplasia progresses, there is an increased probability of malignant transformation into chondrosarcoma [4]. Synchronous multicentric chondrosarcomas arising from Ollier's disease have also been previously reported [4]. The treatment involves correction of angular deformities and limb-lengthening procedures [2].

Radionuclide bone scanning is one method used to assess lesions depicted on radiographs or magnetic resonance images that are presumed to be enchondromas [2]. Also, a bone scan may give a clue to the multifocal nature of the disease.

## Case presentation

**Figure 1:**
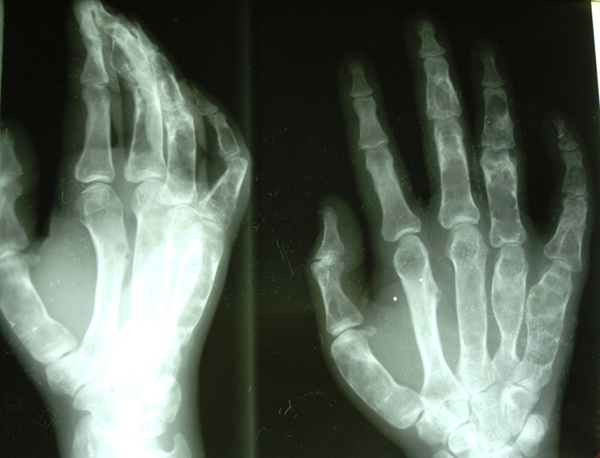
**Bone lesions in the small bones of the right hand consistent with multiple enchondromas**.

**Figure 2:**
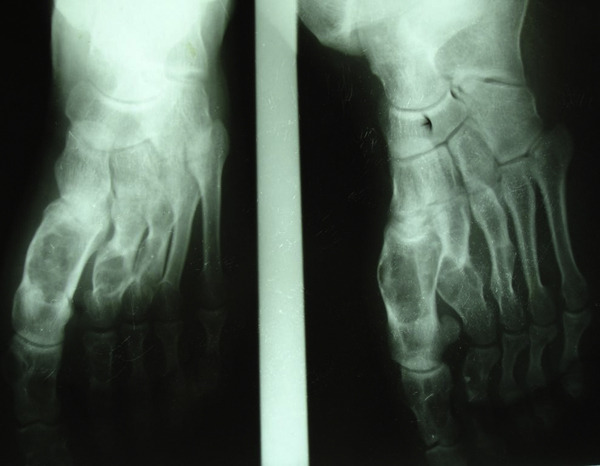
**Bone lesions in the small bones of the right foot consistent with multiple enchondromas**.

A 21-year-old man presented with a history of pathological fractures of the right tibia and multiple incidences of limb surgery related to this rare dysplasia. Radiography assessment showed multiple radiolucent, expansile, homogenous lesions with an oval or elongated shape and well defined, slightly thickened bony margins, suggestive of multiple enchondromas (Figures 1 and 2). There was no cortical erosion, extension of the tumor into soft tissues or irregularity or indistinctness of the surface of the tumor.

**Figure 3:**
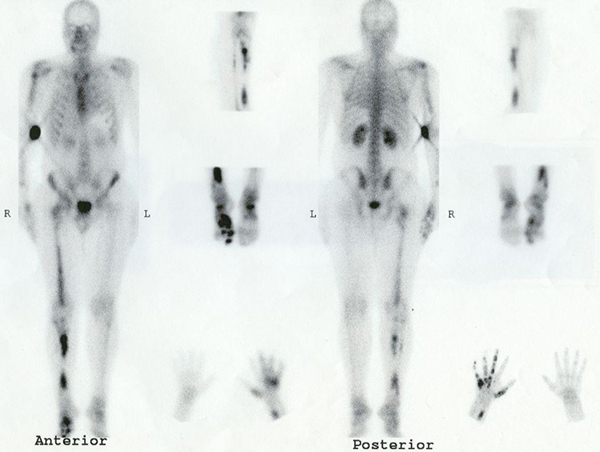
**Bone scan of the same patient showing areas of radiotracer uptake in the humerus, ulna, femur, tibia, metacarpal, metatarsal and phalyngeal tubular bones, all located asymmetrically in the right side of the body**.

A whole-body bone scan obtained 3 hours after intravenous injection of 20 mCi (740 MBq) ^99m^Tc-MDP showed multiple foci of increased activity involving the proximal and distal right femur and tibia, proximal right humerus, distal right ulna, right metacarpals, metatarsals and phalyngeal tubular bones, consistent with a unilateral distribution of the lesions (Figure 3). There were no deformities of the affected limbs. The long bones of the left hemi-skeleton were unremarkable, representing unilateral involvement of the skeleton.

In this case, the intensity of uptake in lesions of the lower extremity was so high that the possibility of malignant degeneration of the enchondromas was raised [4]. Hence, the patient underwent surgical excision of the suspected lesions. Pathology analysis revealed their benign nature.

## Discussion

Chondromas are benign tumors of hyaline cartilage. They may arise within the medullary cavity, where they are known as enchondromas. In fact, enchondromas are the most common cause of intraosseous cartilage tumors. They are most frequent from the 20th to the 40th year of age. The cartilage tumors in enchondromatosis are asymptomatic and are detected as incidental findings. However, the cartilage tumors in enchondromatosis may be numerous and large, producing severe deformities. The radiography features are characteristic, as the unmineralized nodules of the cartilage produce well-circumscribed oval lucencies that are surrounded by a thin rim of radiodense bone (O-ring sign). If the matrix calcifies, it is detected as irregular opacities.

It has been emphasized that Ollier's disease usually stops spontaneously with skeletal maturity; therefore, any lesion showing activity or increased uptake after termination of the growth period requires thorough examination [2,5]. Scintigraphy has been recommended as useful in the monitoring of lesions and of the development of any malignant transformation [5]. Although the malignant transformation of enchondromas is a well-known phenomenon, it should be kept in mind that other etiologies can be considered as the cause of intensely increased uptake.

As was mentioned by Silve and Juppner, the histopathological criteria for malignancy, which are used for conventional chondrosarcomas, cannot be applied for Ollier's disease because of the increased cellularity; hence, distinguishing enchondromas from grade-I chondrosarcomas in the context of enchondromatosis is extremely difficult or even impossible [6]. The diagnosis therefore is based on the combination of radiological, clinical and histological criteria [6]. In our case, as there was no cortical destruction or soft tissue extension, the histopathological diagnosis of benign lesions was reliable.

Retrospective assessment of our patient revealed that the etiology of increased uptake in the lower limb lesions was due to previous insufficiency fractures and the possibility of malignant transformation was ruled out based on the pathology findings.

## Conclusion

Although the malignant transformation of enchondromas is a well known phenomenon, it should be kept in mind that other etiologies should also be considered as the cause of intensely increased uptake.

## Consent

Written informed consent was obtained from the patient for publication of this case report and any accompanying images. A copy of the written consent is available for review by the Editor-in-Chief of this journal.

## Competing interests

The authors declare that they have no competing interests.

## Author's contributions

BF revised the article for intellectual content details and helped to draft the manuscript. AG participated in writing of the manuscript and interpretation of the scintigraphic figures. KA, MB and DB supervised the acquisition process and interpreted the scintigraphic and radiological images. All authors read and approved the final manuscript.
